# Mr. Goodwin, on the Use of the Bark

**Published:** 1800-05

**Authors:** W. Goodwin

**Affiliations:** Earlsham, Suffolk


### *To the Editors of the Medical and Phyfical Journal,*

GENTLEMEN,

HAVING received much pleafure and information from your valuable Journal, I take the liberty of fending a few obfervations on the ufe of the Bark in fome cafes after parturition, which, if you approve of, I fhould be glad you would infert in your next Number. I am,

GENTLEMEN,

Refpectfully, your humble fervant,

W. GOODWIN.

*Earlfham, Suffolk,*

*Feb. 26, 1800.*

---

A few years ago, many lying-in women in this neighbourhood were feized, on the fecond or third day after delivery, with fevere rigor, attended with inflammation, pain and tenfion in the hypogaftric region, and feveral of thefe cafes terminated in death. Moft of thefe unpleafant fymptoms occurred after natural labours, and to women generally healthy. The plan which was adopted, when it was put in practice before a fecond rigor came on, faved feveral; and it confifted in giving the bark freely, and in bliftering the abdomen. The frequency of thefe melancholy cafes, during a feafon, fpread a general alarm over a confiderable diftrict among the fair fex, and put feveral of my medical friends on the practice of giving a decoction of bark with tincture of opium and gum arabic, and fome cordial water or fal volatile, in moft cafes, *immediately* after delivery. Thefe medicines acted as tonics, and leffened or totally prevented the rigor, the confequent fever, and the local inflammation. I hope this practice, now founded on the experience of many eminent and extenfive practitioners in midwifery in this neighbourhood, will appear the more reafonable, when we reflect on the debilitated ftate of many women previous to delivery, and of almoft all of them immediately afterwards, from the lofs often fuftained by hæmorrhage, the great relaxation and proftration of ftrength, and the ichorous and fœtid difcharge. What medicine is fo likely to obviate all the evils attending moft of the cafes after delivery as the bark with opium, and other cordial remedies? It ftruck me very ftrongly, that if Elizabeth Thompfon, whofe cafe was fo ably ftated by the accoucheurs of the Manchefter Hofpital, had taken the bark *immediately* and freely, after the Cæfarean operation, fhe might perhaps, have been faved, to thank her medical friends for their great great humanity and attention, as ſhe appears to have been loſt more from ſphacelus (incipient probably before the operation) than from the operation itſelf.

One lady, for whom I am particularly intereſted, has evidently been ſaved in her laſt three caſes of parturition, by the uſe of the bark; for although ſhe is very healthy when not pregnant, yet as ſoon as ſhe becomes ſo, her appetite declines, her ſtrength and fleſh gradually waſte, ſo that ſhe is nearly reduced to a ſkeleton at the time of her labour. This lady was delivered, on the 19th of February, of her fourth child, and had more hæmorrhage than uſual on the ſeparation of the placenta; but on recurring to my uſual practice with bark and opium, ſhe went on extremely well for twenty-four hours, and I have no doubt would have continued ſo, if the nurſe had not imprudently kept her too hot, which brought on ſyncope and other bad ſymptoms, from which ſhe was recovered by bark, opium, ſal volatile, and red port; and I certainly believe they ſaved her life. Theſe medicines were continued; and during the firſt eight days, ſhe took more than five pints of a mixture of decoction of bark with aromatic tincture and tincture of opium, beſides red port and other good things. She is now recovered, and ſuckles her child, and has had neither rigors nor milk fever, which I am inclined to think may always be obviated, or certainly moderated, by the foregoing method of treatment. Is not this practice likely to leſſen, if not prevent, the wrong action that takes place in the hypogaſtric region after parturition, producing puerperal fever and all its fatal conſequences?

I am now returned from aſſiſting three medical friends, in a long laborious caſe, in a woman of forty years of age, with her firſt child, and in which the crotchet was at laſt obliged to be uſed: It was our unanimous opinion, that no time ſhould be loſt in giving the decoction of bark, tincture opium, &c. and all my friends ſpoke highly of the advantages they had experienced from them in a variety of lying-in caſes. Where there is much hæmorrhage, and a reaſon to apprehend contuſion from the ſeverity of the labour, as well as in all caſes attended with fœtor and debility, the bark with opium are more immediately neceſſary, to which kali, ppt. may often be joined with advantage.

